# Encapsulated Food Products as a Strategy to Strengthen Immunity Against COVID-19

**DOI:** 10.3389/fnut.2021.673174

**Published:** 2021-05-21

**Authors:** Soubhagya Tripathy, Deepak Kumar Verma, Mamta Thakur, Ami R. Patel, Prem Prakash Srivastav, Smita Singh, Mónica L. Chávez-González, Cristobal N. Aguilar

**Affiliations:** ^1^Agricultural and Food Engineering Department, Indian Institute of Technology Kharagpur, Kharagpur, India; ^2^Department of Food Engineering and Technology, Sant Longowal Institute of Engineering and Technology, Longowal, India; ^3^Division of Dairy and Food Microbiology, Mansinhbhai Institute of Dairy and Food Technology, Mehsana, India; ^4^Department of Life Sciences (Food Technology), Graphic Era (Deemed to Be) University, Dehradun, India; ^5^Bioprocesses Research Group, Food Research Department, School of Chemistry, Universidad Autonoma de Coahuila, Unidad Saltillo, Saltillo, Mexico

**Keywords:** bioactive compounds, curcumin, probiotics, COVID-19, SARS-CoV-2, encapsulated food, human immune system, immunological activity

## Abstract

In December 2019, the severe acute respiratory syndrome-related coronavirus 2 (SARS-CoV-2)—a novel coronavirus was identified which was quickly distributed to more than 100 countries around the world. There are currently no approved treatments available but only a few preventive measures are available. Among them, maintaining strong immunity through the intake of functional foods is a sustainable solution to resist the virus attack. For this, bioactive compounds (BACs) are delivered safely inside the body through encapsulated food items. Encapsulated food products have benefits such as high stability and bioavailability, sustained release of functional compounds; inhibit the undesired interaction, and high antimicrobial and antioxidant activity. Several BACs such as ω-3 fatty acid, curcumin, vitamins, essential oils, antimicrobials, and probiotic bacteria can be encapsulated which exhibit immunological activity through different mechanisms. These encapsulated compounds can be recommended for use by various researchers, scientists, and industrial peoples to develop functional foods that can improve immunity to withstand the coronavirus disease 2019 (COVID-19) outbreak in the future. Encapsulated BACs, upon incorporation into food, offer increased functionality and facilitate their potential use as an immunity booster. This review paper aims to target various encapsulated food products and their role in improving the immunity system. The bioactive components like antioxidants, minerals, vitamins, polyphenols, omega (ω)-3 fatty acids, lycopene, probiotics, etc. which boost the immunity and may be a potential measure to prevent COVID-19 outbreak were comprehensively discussed. This article also highlights the potential mechanisms; a BAC undergoes, to improve the immune system.

## Introduction

The increased understanding of the close relationship between food and human health in developed societies has dramatically altered food choice, leading consumers to select one particular foodstuff over another to achieve certain desired health effects (1–3). In this context, functional foods are excellent food choices because they seek to enhance the quality of life by preventing diseases associated with diet (3). Functional foods may be described as foods that offer health benefits beyond their nutritional value such as probiotics, prebiotic compounds, dietary fibers, vitamins, essential oils, etc. As per the CLYMBOL project (the first European Commission-funded cross-country analysis to investigate the positions of public health claims and symbols and to compare food and beverage claims status quo in Europe), these foods that have health associated claims are considered to be marginally safer than other foods without any message (2–4).

**Figure 1:**
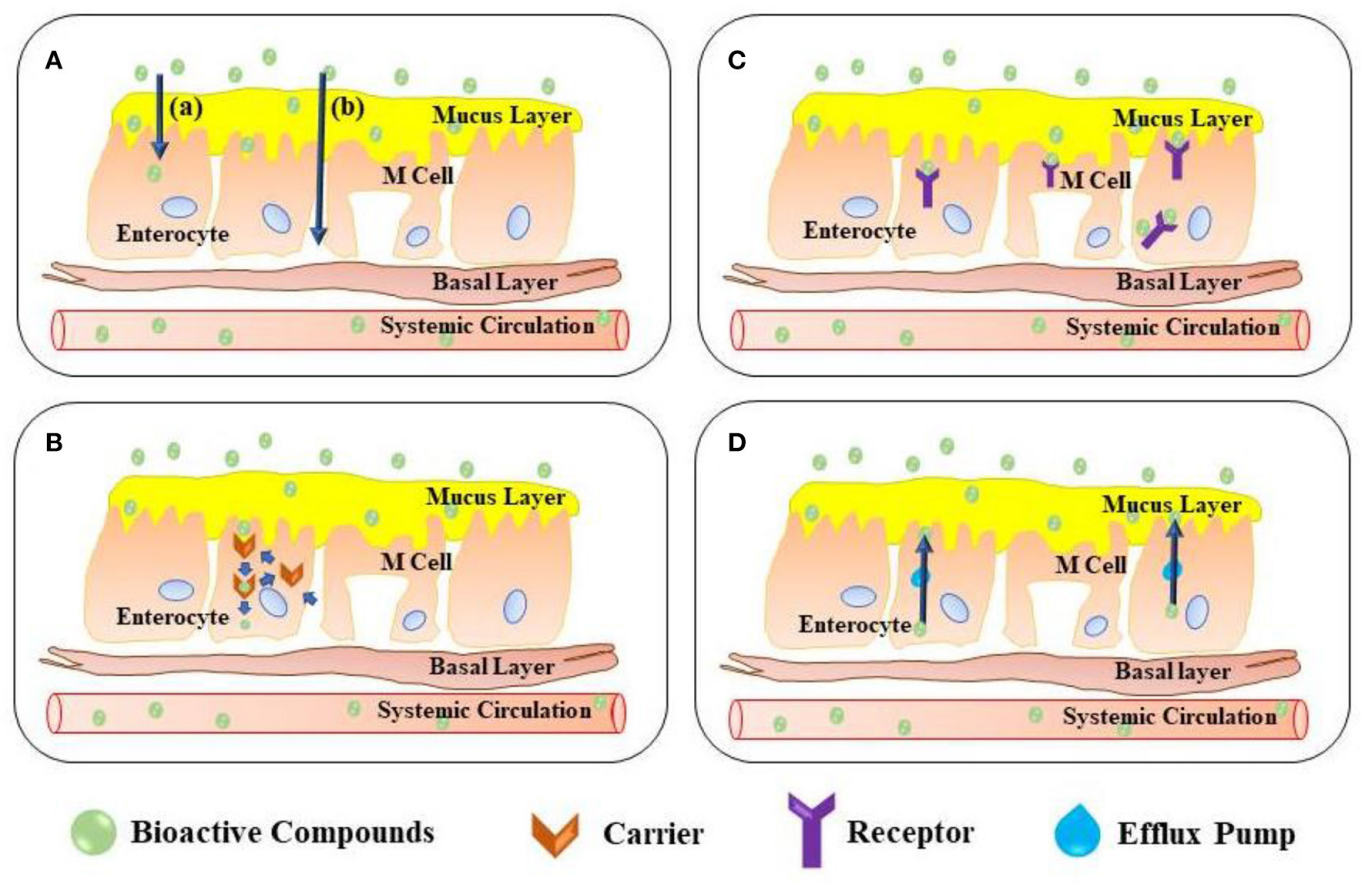
Transport mechanisms for bioactive components through intestinal absorption cells. **(A)** Passive diffusion in transport mechanisms provides an energy-independent route due to osmotic pressure, either by paracellular or transcellular pathway (11). Both pathways defining the mechanism, (a) the pathway of transcellular diffusion in a process where small hydrophobic molecules combine and are then transported into the membrane, while (b) another pathway of paracellular diffusion happens when the junctions of the intestinal epithelium cells are carried by small hydrophilic molecules; **(B)** Transportation mediated by carrier permits the introduction or expulsion of BACs from cells due to osmotic pressure gradients used by cellular protein transporters. These carriers include energy-dependent active transportation and easier diffusion; **(C)** Transportation mediated by the receptor, in which BACs are directly bound and internalized by cells to cell-surface receptors; and **(D)** In the mechanisms of efflux pumps, the most important ATP-based efflux pump of which is a *p*-glycoprotein (*p*-GlyP) in the intestinal epithelium cells. The use of a *p*-GlyP pathway to pump out BACs from enterocytes, leading to limited biological supply. This process has been reported to specifically affect the poor bioavailability of polyphenols.

The food industry and consumers are very involved in the use of functional food as they are believed to improve human health (5). The global functioning food market in 2018 was estimated at USD 161.49 billion and is expected for the next 5 years to rise at a cumulative annual growth rate of 7.9% (6). But the lack of absorption of functional foods limits its health benefits. Water solubility and low stability are mostly attributed to poor absorption in the human gastrointestinal (GI) tract (2). Functional food ingredients must pass through the human GI tract and absorb enteric epithelial cells as they are supplied orally. Low water solubility in the human lumen contributes to low dispersion, resulting in limiting absorption. The extremely low pH (~2.0), on the other hand, of gastric fluid and digestion enzyme can degrade functional food ingredients in the human stomach (7–9). Consequently, many usable food products have very low bioavailability. The carriers based on nanoparticles are therefore very promising to provide bioactive compounds (BACs), as their nanoscale improves enteric epithelial cell absorption (6, 10). A complete pathway and mechanism for the delivery of BACs in intestinal epithelial cells have been shown in Figure 1. The passive transcellular route, paracellular pathway, and endocytosis can be capable of absorbing BAC-loaded nanoparticles (12, 13). Thus, the bioavailability of functionally produced foodstuffs and the benefiting effect of the encapsulating BAC have therefore been expected to increase substantially.

The severe acute respiratory syndrome-related coronavirus 2 (SARS-CoV-2), a new coronavirus appeared in December 2019 and spread rapidly through more than 100 countries worldwide triggering an international outbreak of serious acute respiratory syndrome. On 21 January 2020, coronavirus disease 2019 (COVID-19) was treated by the Chinese Government as infectious diseases of class B and prevention and control as infectious diseases of class A. On 11 February 2020, the World Health Organization (WHO) named the novel coronavirus pneumonia COVID-19. In early August 2020, there were more than 20 million COVID-19 confirmed cases worldwide and more than 728,000 deaths (14). This isn't the first time that coronavirus is causing a major infectious disease. The Coronaviridae family comprised both SARS-CoV in 2003 and the Middle East respiratory syndrome-related coronavirus (MERS-CoV) in 2012 (15). Because of the infectivity of SARS-CoV, SARS-CoV-2, and MERS-CoV, a high fatality rate, and lack of advanced medicine, every single outbreak of coronavirus has been a major burden to society (16). This pandemic of COVID-19 posed several questions about how we can improve the body's immunity through food. SARS- CoV-2 is associated with the invasion and pathogenesis of the host immune system. The first line of defense against invasion is inherent immunity. In innate immunity, mammalian viral infection activates intracellular recognition pattern receptors that sense pathogens of molecular patterns, such as two-strand ribonucleic acid (RNA) or uncapped messenger RNA (mRNA) (17). Several approaches for immunotherapy were previously used in patients with COVID-19 to treat or avoid virus infection (18). Such procedures have been introduced with varying efficiencies in COVID-19, including plasma-recovery therapy, IL-6 monoclonal antibodies, and the C5 protein supplement, cytokine therapy, mesenchymal stem cell therapy, and intravenous immunoglobular disease (15, 19, 20). The virus's interaction with the immune system mediators triggers an immune response that can decide the viral infection outcome (21).

Recent research has shown that dietary supplementation may be effective in patients with COVID-19. Higher than the daily doses of recommended nutritional components such as vitamins (C, D, and E), zinc (Zn), and omega (ω)-3 fatty acids may benefit from reducing the infection rate of SARS-CoV-2 and hospitalization time (22–24). The antioxidant and immunomodulant effects of these nutrients are well-known. Such nutrient deficiencies may lead to immune dysfunction and increase susceptibility to pathological infection. We also analyzed some recently published reports and found that the published reports have described a lack of such nutrition (especially vitamins and minerals) in a diet that makes a significant contribution to the human immune system and therefore undermines immunity. As a result, it may develop morbidity and mortality for a group of elderly people (such as COVID-19 patients) who are at high risk (25–27). However, the elderly are considered to be more likely to be nutrient deficient and less immune, which increases their likelihood of bad COVID-19 results significantly, making proper nutrition double as critical (24). Further, it is essential to take protective measures and keep oneself secure and safe before obtaining a cure and novel coronavirus vaccine. This needs a range of functional food products that boost the immune system and assist in the reduction and alleviation of mortality rates in COVID-19 patients.

Considering such viewpoints, this review explores the importance of encapsulated food products to boost immunity and reports studies about improvement in human health due to encapsulated BACs in the past decades. The present work discusses the overview of existing functional components in different food products and how they can play a role against COVID 19. It also deals with novel encapsulated BACs that have the potential to introduce in food systems to impart immunological activity and develop potential functional foods. Such compounds promote an active immune system and can provide protection against COVID 19. This manuscript is, therefore, an attempt to review the potential of encapsulated functional components to fight against COVID-19 which may support researchers and scientists to develop novel food-based formulation as a preventive measure for COVID-19.

## Human Immunity System, COVID-19 and Functional Foods

Our entire body contains the immune system organ for the defense of disease. Health and pathogenesis have a key role to play. It also protects our bodies from infectious chemicals, germs, and cell changes (neoplasm). White blood cells (WBCs) will pass through the blood vesicles around the body and are the main players in the immune system (28). Our body shares blood and lymph vessels with cells and liquids and helps the lymph system to check for microbial invasion (29). Lymph is stored in the lymph vessels. Specialized compartments for each lymph node can be found in which antigens may be present. The immune cells and foreign particles enter the lymph nodes through the inbound lymphatic vessels (28). They are processed into tissues in the body while they are in the bloodstream. They start throughout the process by targeting foreign antigens and then heading back slowly to the lymphatic system. In the lymph nodes and spleen cells, the immune cells absorb, function, and help to counter antigens (28).

COVID-19 is an approximately 60–140 nm diameter crown-like RNA virus (30). It is spread by coughing and sneezing through respiratory droplets. It reaches our nasal system by inhalation and begins replicating (31). The principal COVID-19 receptor is angiotensin-converting enzyme-2 (ACE2). In the host cell, the spike protein located on the COVID-19 surface is pinched within the ACE2 receiver binding (18). Then, the virus spreads and enters the respiratory tract from the airway. There is the immune response faces a ruggeder innate. The disease is clinically expressed at this level and cytokine may be an unconscious reaction to the subsequent clinical path (32). For 80% of infected patients, the disease is mild and is primarily limited to the upper and leading airways (33). Such individuals can be monitored and controlled at home using conservative symptomatic therapy. Roughly 20 percent of infected patients experience lung infiltration, some of which are very severe (34). Most COVD-19 patients were elderly patients with essential diseases in the extreme category (35, 36). The extreme category was more likely than the moderate to have obstructive pulmonary disorders, asthma, malignant tumor, coronary artery disease, and chronic kidney disease (16, 25, 37). Of those 145 critical patients, 51 were killed; representing 34.69 and 90.2% were dead over the age of 60. Of 51 deaths, 40 patients (78.43%) have a specific illness. Recent studies show patients over 60 years of age with comorbidities, especially hypertension, as risk factors for serious diseases and death of infection with SARS-CoV-2 (16, 36, 37).

**Figure 2:**
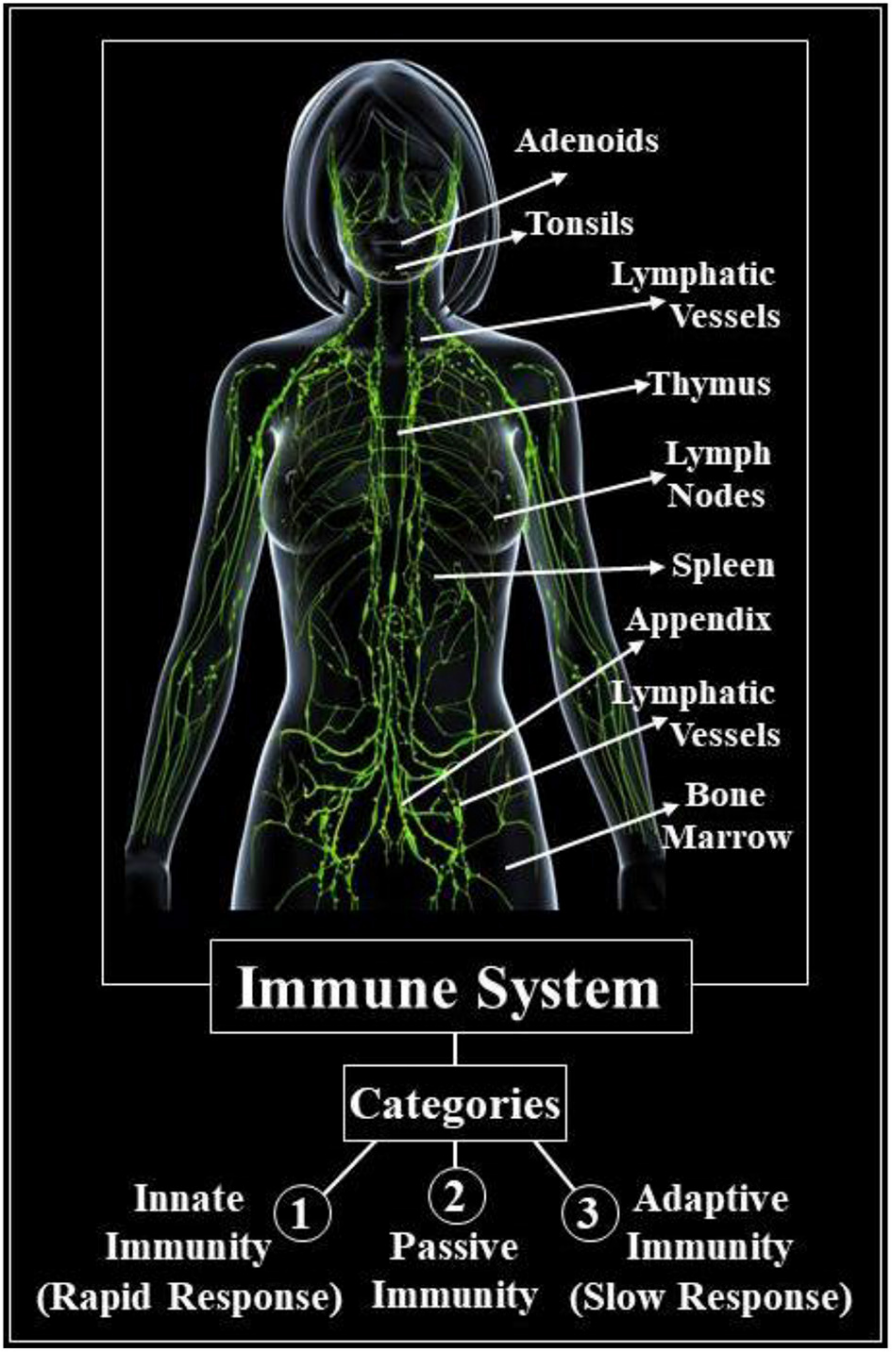
Different categories of the human immune system (28).

The innate immunity mechanism of the human body helps to protect against infectious agents including viruses, bacteria, fungi, protozoa, and worms that resist infections (16, 17). In this scenario, as with other viral infections, our body's immune system can provide us with the best defense against coronavirus since there is no registered COVID-19 medication. We can't note infections like COVID-19 as long as the immune system is strong or active with regard to the development of antibodies and specific immune mechanisms to fight against a specific infectious agent (18). It can be divided into three groups of our immune systems which include (1) innate (or natural) immunity, (2) passive immunity, and (3) adaptive (or active) immunity (as depicted in Figure 2). We are all born with inherent immunity, which serves as a form of general defense. The skin, for example, serves as a shield to prevent black germs from penetrating the body. Two forms of passive immunity are again normal, that is, one in which we obtain artificial immunity from our mother and the second we obtained from drugs or food. The third type, adaptive immunity develops throughout our lives, that is, when we're exposed to diseases or when we're immunized against them with vaccines we develop adaptive immunity. When our body is affected, it starts with skin and inflammatory reactions (21, 22, 28). But the immune system cannot act properly when our body experiences viruses or germs for the first time, and we get sick (16–18). In the case of COVID-19, the same thing happened.

**Figure 3:**
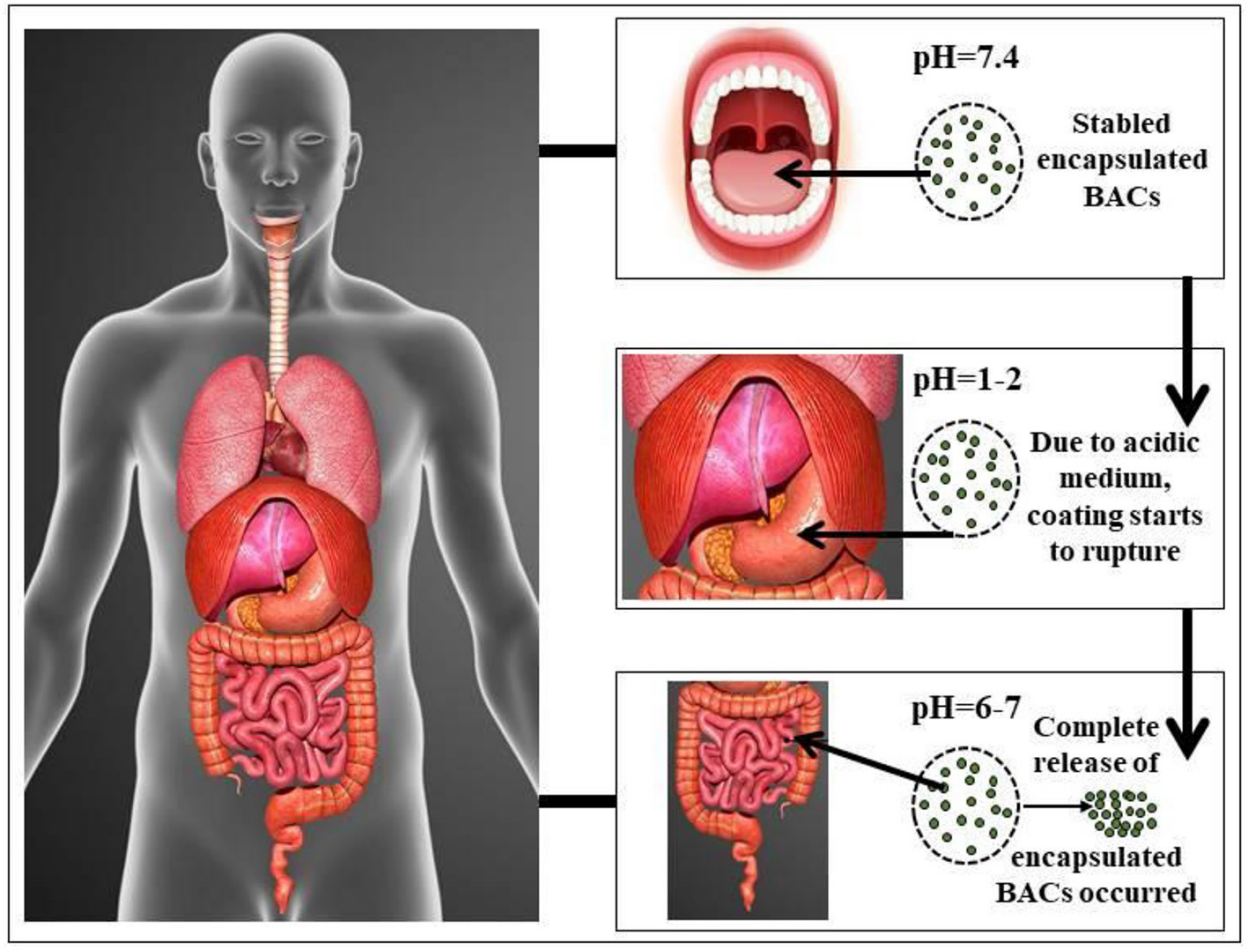
Effect of different pH on the encapsulated bioactive compounds (BACs) in different parts of the human body (11).

Recently, the protective phase that is further divided into two phases in immune defense has been identified as important for COVID-19 treatment and its damaging phase division due to inflammation (38). The damaging phase means when the virus attempts to infiltrate the body's tissues and organs, resulting in inflammation of the particular organ. In the first protective phase, doctors will try to increase immune response and suppress it in the second phase. The improvement in immunity can be accomplished with nutrition supplements containing encapsulated BACs in patients (Table 1) during mild corona infections or by routine intaking of these usable nutrients (25, 35, 39). The WHO has recommended in this regard that balanced foods and hydration are important. People who eat a healthy diet have a good immune system and are at reduced risk of chronic disease and infectious diseases (82). Vitamin and minerals are of essential importance. For example, vitamin-B protects against infection while vitamin-C protects us from signs of flu. In the case of coronavirus infection, it has been observed that the incorporation of half tea spoon turmeric (curcumin) powder in hot milk cures sour throat and boosts immunity, and hence it is also recommended by The Ministry of AYUSH. Similarly, use of various herbs like Tulsi (Basil), Kalimirch (Black pepper), Dalchini (Cinnamon), Giloy (*Tinospora cordifolia*), and Shunthi (Dry Ginger) in hot water as kadha as well as lemon juice (a rich source of vitamin-C) had also shown to improve the immunity against COVID-19 virus (83–86). The active compounds from such foods can be extracted, encapsulated, and used to treat COVID-19 (24, 66, 87). However, currently, all these work is at a nascent stage and it may further demand proper systematic approaches to prove the positive effects on the immune system. Vitamin-D or vitamin-E deficiency may contribute to coronavirus infection. But we can get these vitamins from their natural sources to overcome the problem (24, 88). For example, sunlight can provide vitamin-D, while oil, seeds, and fruits can provide vitamin-E. Failure to produce iron (Fe) and excess of Fe may lead to risk (89). While, the maintenance of our immune system requires Zn (24, 90). Protein-rich food should be at the forefront because the food has immune properties (production of immunoglobulin) and possible antiviral activity. Therefore, whole cereal grains, legumes, vegetables, fruits, nuts, and animal food should be consumed on daily meals. However, these biologically active compounds are very sensitive to the low pH of the belly and are not of great health benefit to our body because of their poor bioavailability in our GI tract (11, 91, 92). As a result, the encapsulation of a biological compound with immunological activity increases the safety of BACs in low pH conditions and increases their bioavailability in the GI tract. Figure 3 showed the effect of the pH of the mouth, stomach, and intestine on the encapsulated particle and described how the encapsulated particle of the BAC can protect at lower pH of the stomach (11). The use of these encapsulated compounds in the preparation of functional food is a novel method for improving the functionality of food as well as for enhancing human immunity to various microorganisms, such as the novel coronavirus. According to a recent survey of the Food Safety and Standards Authority of India (FSSAI), sales of immunity-boosting foods have increased by about 20–40% since the country went into lockdown due to the COVID-19 pandemic (93).

**Table 1:** Different bioactive compounds (BACs) having immunity-enhancing activity.

**Compounds**	**Role in action mechanism**	**References**
Omega (ω)-3 fatty acid/polyunsaturated fatty acid (PUFA)	Affect both immunity systems, innate as well as adaptive	(24, 39–41)
β-carotene	Boosts the immune response in all cells and humor in both laboratory animals and humans	(42, 43)
Carvacrol	Affect neutrophil and lymphocytes	(44, 45)
Curcumin	Improve immunoglobulin (IgG and IgM) levels and WBC (white blood cells) values	(46)
Mangiferin	Activate lymphocytes, neutrophils, and macrophages	(47, 48)
Resveratrol	Multiple immune responses and signaling pathways. Moreover, it suppresses pro-inflammatory genes' expression and the toll-like receptor (TLR)	(49–51)
Thymol	Reduces the T-cell over activity in immune-mediated diseases	(52)
Vitamin-A	Regulate cellular and humoral immune processes	(53, 54)
Vitamin-B	Have interactions with immune cells involved in inflammation as well as pathophysiological pathways	(55, 56)
Vitamin-C	It includes the immune defense of the innate as well as adaptive immune systems by promoting the cellular function	(24, 57–60)
Vitamin-E	It acts by antioxidant routes, which include increasing the amount of T-cells, improving mitogenic lymphocyte reactions, increasing the secretion of IL-2, and increasing the activity of NK cells	(24, 58, 61–63)
Vitamin-D_3_	Vitamin-D has an immunomodulatory function, enhancing innate immunity through the antiviral peptide portion, enhancing mucous protection	(24, 64, 65)
Copper (Cu)	It is involved in the functions of different types of cells, including B-cells, T helper cells, natural killer cells, and macrophages	(66, 67)
Iron (Fe)	Involved in the innate immune system	(68, 69)
Magnesium (Mg)	Plays an important role in both immune responses, both innate and adaptive	(66, 70, 71)
Selenium (Se)	Works through anti-oxidant pathways to increase T-cell levels, promote mitogenic lymphocyte reactions, increase the secretion of IL-2, and improve the activity of NK cell	(24, 72, 73)
Zinc (Zn)	It also plays an important role in both innate and adaptive immune responses	(24, 74–76)
Probiotics and Prebiotics	Different probiotics, prebiotics, and/or combinations of both have important impacts on the immune system and various immune networks of host immunological systems	(77–81)

## Encapsulated Food Products as a Potent Immunity Booster

**Figure 4:**
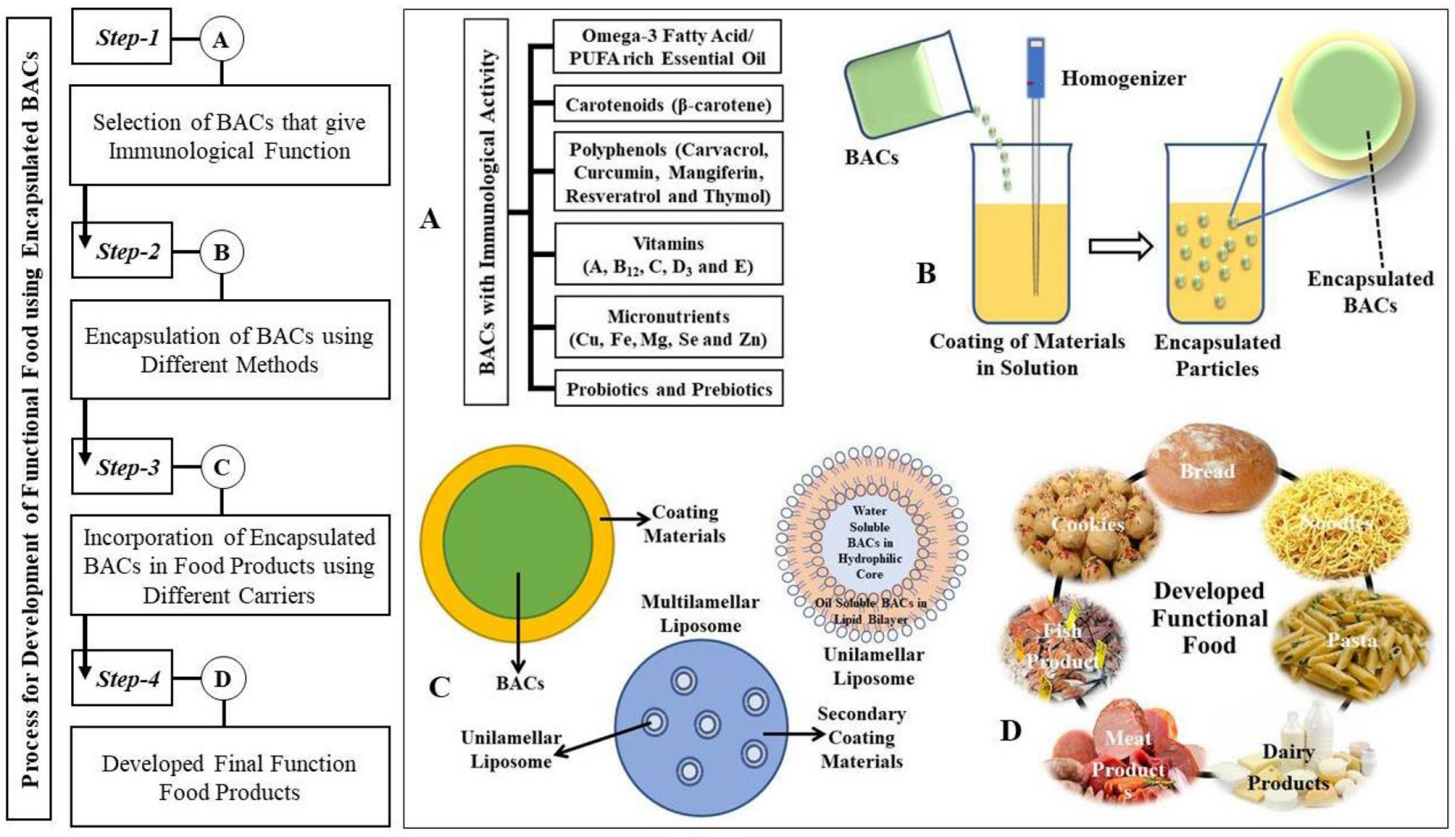
A general flow schematic diagram for the development of functional food using encapsulated bioactive compounds (BACs) of immunological activity (96). **(A)** First, food-grade BACs that are supposed to substantially increase immunological function are chosen. **(B)** Second, nanoparticle-based carriers are used to encapsulate the BACs since they have shown promise in delivering BACs. Since functional food ingredients are delivered orally, they pass through the human gastrointestinal (GI) tract and are absorbed by enteric epithelial cells, which is why nanoparticle-based carriers are used to deliver BACs. However, low water solubility in human lumen fluid, results in low dispersal, reducing absorption. On the other hand, certain functional food additives may be degraded by an extremely low pH (~2.0) of gastric fluid and digestion enzymes in the human stomach. **(C)** Following that, these encapsulated BACs are incorporated into food formulations through various carriers to develop formulated functional food. **(D)** Thus, by using these evolved final encapsulated functional foods, we may be able to improve the immunity of the human body against the novel Covid-19 virus.

The demand of consumers for healthier natural food products has increased in recent years and has shown scientific effectiveness in finding nutrients in alternative food sources (1, 3, 94). Inadequate nutrition, caused by eating, led to a widespread incidence of diseases such as obesity, cancer, cardiovascular disorders, or immunological diseases. This created an ideology of the modern consumer who wants food that has the ability to protect your health and avoid disease (95). The food industry is therefore centered on the production of functional foods with natural compounds that provide health benefits, such as antioxidants, anti-inflammatory activity, or immunological behavior. A schematic diagram for developing various functional foods is illustrated in Figure 4 that describes the encapsulation of the immunologically active BACs. These functional foods are known as nutraceuticals as such foods can help to cure diseases caused by imbalanced feeding or avoid them. The word “nutraceutical” is a nutrient-pharmaceutical fusion, first introduced in 1989. It is considered a good diet capable of preventing illness which removes the obstacle between food and medicines and which clearly demonstrates that food of this sort provides an incredible potential to enhance human health (2, 3, 95).

### Cereal-Based Products

Bioactive compound encapsulation retains the compound's structure and functional quality and has a limited effect on the organoleptic perception of food products, including bread in food matrix form (97, 98). The encapsulated material and the BAC contain the capsular wall material of the sample matrices, which provides a barrier between the BAC and the bread dough (98). The encapsulating cell wall materials typically are starch, starch derivatives, lipids, proteins, gums, and all combinations thereof. Spraying and freeze-drying techniques for encapsulation of BACs for bread making are the most common and relatively simple (97). Table 2 shows the wall materials used to encapsulate the functional ingredients used in the formulation of bread. These materials have been used by many people for the favorable effects of bread quality.

**Table 2:** Wall materials for functional ingredients encapsulation.

**Wall Materials**	**Abbreviations**	**References**
**Single form**		
Alginate		(99)
β-cyclodextrin	CD	(100)
Calcium-gelatin casein	CGC	(101)
High amylose corn starch	HACS	(102)
Maltodextrin	MD	(97, 100)
Methylcellulose	MC	(101)
Soybean protein isolates	SPS	
Whey protein concentrate	WPC	
Whey protein isolate	WPI	(97)
**Combined form**		
Maltodextrin + β-cyclodextrin	MD + CD	(100)
Whey protein isolate + maltodextrin	WPI + MD	(97)

**Figure 5:**
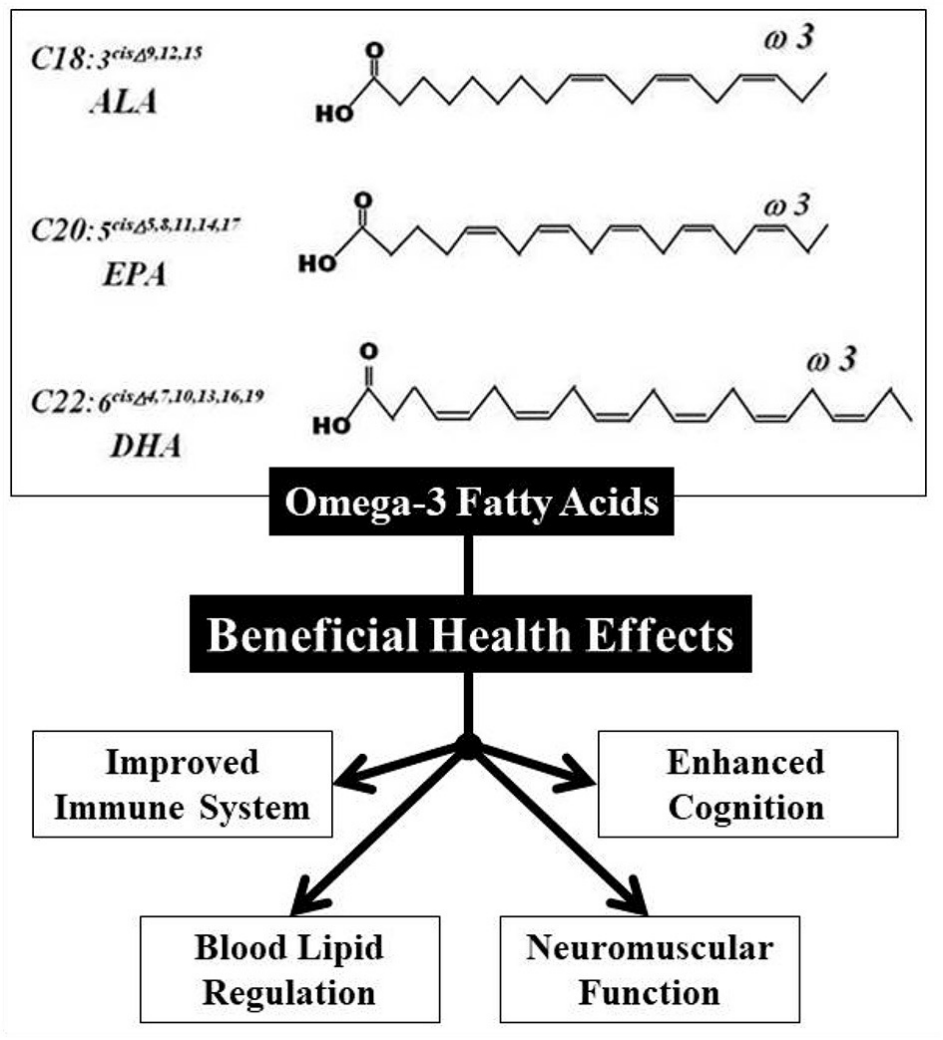
Different health benefits of omega (ω)-3 fatty acids in the human immune system (157–159).

Different compounds such as ω-3 fatty acid, *n*-3 polyunsaturated fatty acids (PUFAs), curcumin, and probiotic bacteria have been encapsulated and used for the production of cereal-based functional and/or fortified food (Table 3A). Omega (ω)-3 fatty acids, however, are a class of long-chain acids that have demonstrated various beneficial health effects (Figure 5) (157, 158). Omega (ω)-3 fatty acids have recently been encapsulated in soybean phospholipids by liposome technique in order to mask the flavor of fish and used in the making of bread to provide ω-3 fatty acids to the health beneficiary (104, 105). Baking time and temperature were ranged between 13–26 min and 200–260°C, respectively. The sensory assessment showed that enriched bread had a higher amount of nano-liposomal ω-3 fatty acids than other samples. Additions of nano-liposomal ω-3 fatty acids had no detrimental effects on the texture property and sensory acceptability of bread (104, 105). Omega (ω)-3 fatty acids have helped to increase the immunity of the human body by affecting T-cells, macrophages, and neutrophils of the human body (40). T-cells are lymphocytes originating from the thymus that recognize the T-cell receptor (TCR) presented antigens. The association of a TCR with antigen-presenting cells (APCs) such as macrophage or dendritic cells (DCs) is the first time and the T-cells are activated. Alterations in APCs activation using ω-3 fatty acids are also the first pathways for modulating T-cell activation *in-vivo* through the use of ω-3 fatty acids (160). The principal adaptive branch lymphocytes of the immune response are B-cells, along with T-cells. As part of the innate immune system, macrophages play an important role. Omega (ω)-3 fatty acids induce important changes to macrophage gene regulation. The treatment of docosahexaenoic acid (DHA) or eicosapentaenoic acid (EPA) macrophages results in significant changes in the gene expression of lipopolysaccharide (LPS)-activated macrophages derived from T-helper type (THP) 1 (159). However, DHA and EPA do not have the same results. In addition to the cell culture medium for macrophages, ω-3 fatty acids (DHA and EPA) induce essential global changes in the microRNA (miRNA) profile. Neutrophils are the first cells in which inflammation occurs and play a significant role in removing pathogens (161). Neutrophils can communicate with the adaptive immune system, however, through the incorporation of naïve T-cells into THP-1 cells, and can have B-cell antigens in the spleen. After absorption of the ω-3 fatty acids into the phospholipids, they may be metabolized by neutrophils into leukotrienes, maresins, prostaglandins, protectins, resolvins, and thromboxanes (40). The neutrophil role of ω-3 fatty acids and their metabolites is modulated in many ways, they are as follows: phagocytic capacity, cytokines, neutrophil migration, and reactive oxygen species (ROS) production. Omega (ω)-3 fatty acids successfully integrate and control all immune cells into the cell membrane by *in vitro* stimulation or dietary supplementation (40). Successful encapsulation of ω-3 fatty acids has been described in various reports on the development of different food products, such as breads (103), cookies (106, 107) and pasta (114). As ω-3 fatty acid has an immunity-enhancing function, these food items based on cereals can be eaten to improve the body's immunity to foreign compounds that can help to fight against the novel coronavirus (24).

**Table 3:** Food product developed by the encapsulation of bioactive compounds (BACs) containing immunological activity.

**Developed food product**	**Encapsulation matrix**	**Encapsulated compound**	**Method of encapsulation**	**Remark**	**References**
**(A) Cereal-based product**					
Bread	Soy protein isolate	ω-3 fatty acid rich chai oil	Freeze drying	Baking time-15 min and baking temperature-220°C	(103)
Bread	Soy lecithin	ω-3 fatty acid rich chai oil	Liposome	Baking time-13 min and baking temperature-260°C	(104)
Bread	Soybean phospholipid	ω-3 fatty acid	Liposome	Baking time-26 min and baking temperature-00°C	(105)
Cookies	Sodium caseinate, fish gelatin, and glucose syrup	Polyunsaturated fatty acids (PUFAs) rich shrimp oil	Spray drying	Baking time-20 min and baking temperature-205°C	(106)
Cookies	Whey protein concentrate	ω-3 fatty acid rich Garden cress (*Lepidium sativum*) oil	Spray drying	Baking time-8 min, baking temperature-205°C, and a better sensory score of color, crumb color, and surface characteristics of biscuits with microcapsules	(107)
Bread	Arabic gum, maltodextrin, methylcellulose, and WPI	ω-3 fatty acid rich linseed oil	Spray drying	Baking time-20 min, baking temperature-220°C, and poor oxygen permeability by Arabic gum microparticle which minimized lipid oxidation	(108)
Bread	WPI, inulin, pectin, fresh agave sap, carboxymethylcellulose and starch	*Lactobacillus acidophilus*	Spray drying	Baking time- 16 min and baking temperature-180°C	(109)
Cookies	Mesquite gum, maltodextrin and zein	Flavan-3-ol rich grape seed extract	Spray drying	Baking time-8 min, baking temperature-180°C, and 60% customer acceptability to buy cookies	(110)
Bread	–	ω-3 fatty acid	–	Baking time-20 min, baking temperature-160°C, and sensory acceptance for fortified bread	(111)
Bread	Gelatin and porous starch	Curcumin	Spray drying	Increased curcumin bioavailability	(112)
Bread	–	*n*-3 PUFAs	–	Baking time-30 min, baking temperature-180°C, high (80–89%) recovery of DHA and EPA, lower lipids oxidation in bread after baking and storage, and quite stable microencapsulated *n*-3 PUFA powder in bread	(113)
Pasta	Corn starch	*n*-3 PUFA	–	Increased pasta storage	(114)
Bread	Methylcellulose, soybean protein isolates, calcium gelatine casein and whey protein concentrate	ω-3 fatty acid rich fish oil	Spray drying	Baking time-30 min, baking temperature-180°C, and better sensory score for microencapsulation with methylcellulose and soybean protein isolates	(101)
Bread	–	ω-3 fatty acid rich oil	–	Baking time-20 min and baking temperature-220°C	(115)
**(B) Fruit and vegetable-based products**					
Cantaloupe juice	Starch octenyl succinate (starch-OS)	Nisin and Thymol	Emulsion	Better retention of nisin and thymol in emulsions during storage, and greater inhibition of *Listeria* and *Salmonella* than non-emulsion, aqueous formulations	(116)
Fresh apple juice	Ethyl butyrate, Tween 80, and PEG 400	Ascorbic acid (vitamin-C) and vitamin-E	Microemulsion	Decreased brownness and increased shelf-life	(117)
Carrot juice	Modified maize starch	Carvacrol	Emulsion	Decreased harmful microorganism	(118)
Apple juice	Ethyl butyrate, Tween 80, and PEG 400	Ascorbic acid (vitamin-C)	Microemulsion	Decreased brownness	(119)
Mulberry, Maoberry, Longan, and Melon juices	Sodium alginate solution, cashew flower extract, or green tea extract	*L. casei* 01, *L. acidophilus* LA5 and *Bifidobacterium lactis* Bb-12	Extrusion	Increased shelf-life of juices	(120)
Pineapple juice	Sodium alginate solution, oligosaccharides extract	*B. longum* and *Eleutherine americana*	Extrusion	Better sensory acceptability of products with co-encapsulated bacterial cells than free cells	(121)
Orange juice, Pineapple juice, White grape juice	Legume protein, Tween 80	*B. adolescentis*	Emulsion	Survival of encapsulated *B. adolescentis* cells in pineapple and white grape juice, and increased storage	(122)
Carrot juice	Sodium alginate, chitosan	*L. casei* 01	Spray drying	Improved functionality of carrot juice containing *L. casei* and good to lactose-intolerant people	(123)
Apple juice	Resistant starch aqueous dispersion, WPI	*L. rhamnosus*	Spray drying	Better shelf-life at 4°C	(124)
Pomegranate juice	Sodium alginate	*L. plantarum*	Extrusion	Shelf-life up to 6 weeks at 4°C	(125)
Apple juice	Di-palmitoyl phosphatidylcholine (DPPC), Cholesterol	Ascorbic acid (vitamin-C)	Liposome	Enhanced stability of AA in apple juice	(126)
Tomato seeds	Alginate	*B. subtilis*	Extrusion	Fermentation of immobilized cell and better overall palatability in tomato juice than that of free cells in cold storage	(127)
Orange juice	Soy phosphatidylcholine, stearic acid, calcium stearate	Vitamin-E and vitamin-C	Liposome	No alterations in sensory characteristics for the combination of liposomal formulations and vitamins with orange juice, and better microbiological stability after pasteurization and storage at 4°C for 37 days	(128)
Tomato juice	k-Carrageenan	*L. acidophilus*	Extrusion	Better overall palatability for microencapsulated tomato juice	(129)
**(C) Dairy-based product**					
Skim milk	Soy lecithin, glycerol	ω-3 fatty acid rich shrimp oil	Liposome	No major quality changes during the storage of skim milk fortified with shrimp oil nanoliposome at 4°C for 15 days	(130)
Milk	β-lactoglobulin, chitosan	Mangiferin	Extrusion	Higher antioxidant capacity, better inhibition of lipid peroxidation, and protein oxidation in mangiferin nanoparticles fortified dairy beverage	(131)
Yogurt	Lecithin, glycerol	Vitamin-D_3_	Liposome	–	(132)
Lassi (A milk-based Indian beverage)	Monegyl Caprylic/capric triglyceride (CCTG)	Vitamin-D_3_	Liposome	Sensory acceptance of Lassi fortified with vitamin-D_3_ nanoparticles	(133)
Milk	Soybean oil, Tween 20, Lecithin	Vitamin-D_3_	Emulsion	Fortification of whole-fat milk with vitamin-D_3_ enriched nanoemulsions showed stable nature to particle growth and gravitational separation for a minimum of 10 days	(134)
Yogurt	Soy lecithin, sunflower oil	ω-3 fatty acid rich fish oil	Liposome	Similar sensory properties of yogurt containing nano-encapsulated fish oil than control samples	(135)
Milk	Labrafac, lecithin	Vitamin-D_3_	Liposome	Potential usage of vitamin-D_3_ loaded lipid nanocapsules to develop fortified milk	(136)
Yogurt	Whey proteins concentrate (WPC), sodium caseinate and lactose	ω-3 fatty acid rich flaxseed oil	Spray drying	A potential delivery system of ω-3 fatty acids by incorporating flaxseed oil microcapsules in yogurt	(137)
Yogurt	Sorbitan monooleate lauryl alcohol or 1-dodecanol, Polyglyceryl-3 Dioleate, Glycerol monooleate	Iron	Niosome	A little effect on sensory, rheological, and stability properties of control yogurt by iron-entrapped niosomes	(138)
Yogurt	Whey protein isolates, Carboxymethylcellulose	Vitamin-D_3_	Emulsion	Stabilized emulsions as an efficient delivery system of vitamin-D_3_ in fortified yogurt	(139)
Yogurt	Whey protein isolates	Iron	Hydrogel	Similar sensory quality attributes of yogurt fortified using WPI-Fe particles than control samples	(140)
Cheese	Whey protein concentrate	ω-3 fatty acid	Emulsion	–	(141)
**(D) Meat-based product**					
Fish sausage	Canola oil, Tween 80	Tocopherol (vitamin-E)	Emulsion	Delayed lipid oxidation and improved quality without altering texture properties in fish sausages containing encapsulated tocopherol during cold storage	(142)
Fish sausage	Soy isolate protein	ω-3 fatty acid rich cod liver oil	Emulsion	Better textural properties including hardness and decreased springiness in gelled-emulsified fish oil-based sausages	(143)
Chicken nuggets	Chitosan, Tween 80	ω-3 fatty acid	Layer-by-layer deposition technique	Delayed lipid oxidation and microbial spoilage, higher sensory quality, and overall acceptability by addition of encapsulated fish oil during refrigerated storage	(144)
Sausages	Lecithin, chitosan	ω-3 fatty acid	Emulsion	Increased quantity of EPA and DHA by fish oil microcapsules, without influencing physico-chemical properties, oxidative stability, or acceptability	(145)
Deer pâté	Sodium caseinate and lactose	ω-3 fatty acid rich Chia oil, linseed oil, Tigernuts oil	Spray drying	Modification in fatty acid composition of pâtés with microencapsulated oils; decreased amount of SFA and increased levels of PUFA (chia and linseed pâtés) or MUFA contents (tigernut pâtés)	(146)
Chicken meat	Whey protein concentrate, sodium alginate, maltodextrin	ω-3 fatty acid rich flaxseed oil	Emulsion	Detection of greater ω-3 incorporation with higher content of its derivatives and a favorably lower ω-6/ω-3 in broiler meat fed with nanoemulsions containing flaxseed oil	(147)
Cinta Senese pork burgers	Soy lecithin, chitosan and maltodextrin	ω-3 fatty acid	Spray drying	Encapsulated ω-3 showed best scores at chilled condition than control and bulk fish oil added burgers	(148)
Beef burger	Sodium alginate	ω-3 fatty acid rich Chia oil	Ionic gelation technique	Greater oxidative stability in burgers produced with chia oil microparticles enriched with rosemary by ultrasound-assisted extraction	(149)
Chicken nuggets	Lecithin and chitosan	ω-3 fatty acid rich Cod liver oil	Spray drying	No difference in sensory properties between microencapsulated fish oil nuggets and control ones	(150)
Chicken sausages	Soy protein concentrate, gelatine	vitamin-E and ω-3 fatty acid rich flaxseed oil	Spray drying and Freeze-drying	Retention of α-linolenic acid and α-tocopherol in fortified formulations	(151)
Frankfurter sausage	Sodium caseinate	ω-3 fatty acid rich fish oil	Emulsion	Encapsulated batches presented the highest L* and b* values, and non-encapsulated oil treatments showed maximum a* values	(152)
Chicken nuggets	Lecithin-chitosan and maltodextrin	ω-3 fatty acid rich fish oil	–	Protective effect against lipid and protein oxidation, especially during 1^st^ month of storage	(153)
Pork sausage	Konjac gel. maltodextrin, gum arabic and caseinate	ω-3 fatty acid rich fish oil	Spray drying	Increase of hardness, gumminess, and chewiness and decreased the fat content (30.4%) on the incorporation of microencapsulated fish oil without affecting springiness and cohesiveness	(154)
Beef burger	–	ω-3 fatty acid rich fish oil	–	Increased PUFA content and decreased hardness in burgers	(155)
Meat batter	Gum Arabic	*Aerococcus viridans* UAM21, *Enterococcus faecium* UAM10a, *L. plantarum* UAM17, and *Pediococcus pentosaceus* UAM11	Spray drying	Use of thermotolerant LAB as bioprotective cultures to improve safety in cooked meat products, enhancing the nutritional values without any detrimental effect on textural or physicochemical properties	(156)

*The color parameters of a food product.

L*, a*, and b* is the representation of color of the sample.

Besides ω-3 fatty acids, the probiotic microorganisms are being encapsulated for developing novel functional foods with improved functionalities (162, 163). Recently, Muzzafar and Sharma (164) developed the biscuits containing microencapsulated probiotics whereas functional bread was prepared by Seyedain-Ardabili et al. (165) which contained encapsulated strains of *Lactobacillus casei* 431 and *L. acidophilus* LA-5. Bacteria found in the intestines help to digest food and suppress harmful bacteria, increase nutrition, and promote immunity. Several studies have shown that probiotics activate innate and acquired immune responses by stimulating immunoglobulin A (IgA) secretion, encouraging phagocytosis, altering T-cell reactions, and preserving THP-1 and THP-2 homeostasis by improving THP-1 responses and attenuating Th2 responses (166, 167). The mechanism of improving immunity by probiotics in the human host is complex and not thoroughly explained. However, it is assumed that probiotics will facilitate the production of bacteriocins and short-chain fatty acids, reduce the intestinal pH, provide colon nutrients, colonize and participate in binding sites of intestinal epithelial cells, promote mucosal barrier function, and modulate the immune system (168, 169). Microencapsulation is necessary to enhance process efficiency, solubility, dispersibility, flowability, and shelf-life by preventing certain reactions (oxidation, dehydration), improves process protection and convenience, and protecting unstable sensitive compounds (108, 170, 171).

Therefore, it can be suggested to use nano-liposomal ω-3 fatty acids as well as encapsulated probiotics for the enhancement of the nutritional value of bread, biscuits, and other bakery items. Such encapsulated bakery products are the need of the hour to promote the immune system of the human body because the process of encapsulation enhances the viability and thermal resistance of probiotic bacteria and other heat-sensitive compounds.

### Fruit and Vegetable-Based Products

The worldwide consumption of fruit and vegetable juices is widespread. There is a continued increase in world demand for high-quality fresh juices; in particular, as there is a growing awareness that healthy fluids and juices are included in the human diet instead of sweetened beverages and soft drinks. Fruit and vegetable juices are naturally rich in BACs that are major contributors to human nutrition and have health-promoting and disease prevention properties (172). These natural BACs offer a wide range of health benefits to consumers, including bone-protection, skin-protection, cardiovascular disease, normal blood pressure maintenance, nervous system, weight loss, etc. (173–175). However, no evidence has been found that fruit and vegetable juices have been immunologically active so far. The use of natural additives to enhance the immunological activity of fruit and vegetable juices has therefore been extensively made. Antimicrobial products such as essential oils and their components as well as vitamins and probiotics have been reported as natural additives for juice (Table 3B).

Vitamin-C is a water-soluble vitamin (also known as ascorbic acid) that cannot be synthesized in the human body. Vitamin-C is well-known to have protective value in infectious diseases. It is an essential co-factor to form the cartilage, blood vessels, muscle, and collagen in bone and is important for the healing process (176). As an anti-oxidant, vitamin-C protects the cells from damage by free-radicals and contributes to the prevention of stroke, cancer, and other diseases. In reality, supplements are known to aid mechanisms for respiratory defense, prevent virus infections, and reduce their duration and severity, as well as improve flu-like symptoms with anti-histamine properties. It is therefore an important element in COVID-19 because it has the main respiratory defense function (24). A recent study showed an increased high sensitivity C-reactive protein (hs-CRP), a marker of inflammation, and oxidative stress in 29 patients with COVID-19 pneumonia. Transcription factor, nuclear factor erythroid 2 (nfe2)-related factor 2 (Nrf2), is a major regulator of antioxidant response element (ARE)-driven cytoprotective protein expression (177). Activation of Nrf2 signaling is vital for preventing the cells and tissues from injury induced by oxidative stress. Vitamin-C is an essential part of the cellular antioxidant system. Cytokine storm rises as the disease progresses during COVID-19 infection, and vitamin-C against this has been suggested (23, 57). The levels of the pro-inflammatory cytokines, including tumor necrosis factor-α (TNF-α), are known to be reduced and anti-inflammatory cytokines (IL-10) increase (178). After 5 days of production of acute respiratory distress syndrome (ARDS), a study recently published by Khan et al. (23), which describes a patient treated with high-dose vitamin-C, was removed from ventilation and this was exceptionally early. However, they have obtained anti-viral drugs which should be noted (23). The preventive role of vitamin-C assists in scavenging activity of dead cell, vitamin-E regeneration, normal neutrophils function, activating signaling cascade and proinflammatory transcription factor NFκB (nuclear factor kappa B), modulating the signaling pathways, regulating inflammatory mediators, phagocytosis, gene regulation, and signaling pathways in T-cells, and improves the neutrophils motility to reach the infection site. Using such mechanisms, vitamin-C exhibits a promising role in immune modulation and proper immune functions (179–182). Epidemiological studies have also been indicated that vitamin-E deficiencies affect immune responses and infectious diseases (35, 183). Vitamin-E is used to increase the number of T cells, increase lymphocyte mitogenic reactions, increase the secretion of IL-2 cytokine, boost NK cell function and reduce the risk of infection (35, 183). It also shows the suppressing effect on the generation of prostaglandin E2, a T cell-suppressing lipid mediator, which increases with aging. Vitamin-E-induced improvement in the immune system is of important therapeutic effect, as is seen in the results of improved respiratory resistance in both older mice and older adults (183, 184). Vitamin-C and vitamin-E can be supplied to the body using fruit juice (particularly fortified apple juice) that has been prepared by Tao et al. (117). By adding encapsulated vitamin-C (0.05%) and vitamin-E (0.05%) to the juice, the authors created the enhanced apple juice. Vitamin encapsulation was performed in microemulsion methods Ethyl butyrate, Tween 80, and PEG 400. Adding vitamins also decreased the browning and the acceptability of apple juice (117). The frequent use of high vitamin-C and vitamin-E apple juice will improve our body's immunity to viral infections such as COVID-19.

A potential intervention to improve outcomes, particularly for six ventilation-associated pneumonia, probiotics have been recommended in COVID-19-infected patients, in addition to vitamins (185). *Lactobacilli* spp. (120, 123, 124), *Eleutherine* spp. (121), and *Bifidobacteria* spp. (120–122) are the most commonly used probiotics in functional and other fermented foods (162). Thus, there is increasing interest in the development of fruit-juice-based probiotic products (186, 187). Fresh fruits and vegetables are known to be healthy matrices that may be suitable substrates for probiotics because of their antioxidants, fibers, minerals, and vitamins and their existence does not involve any dairy allergens which can prohibit their use by unique segments of the population (188). Fruit juices and beverages have an excellent flavor for all ages and are considered to be safe and healthy. A variety of fruit and vegetables like apples, banana, blackcurrant, oranges, blueberry, cashew apple, pineapple, raspberry, pomegranate juice, cantaloupe melon, pineapple, etc. and mixed vegetable juice are explored for the development of probiotic-containing juices (77, 186, 189–191).

It is well-known that probiotics improve the immunity of humans through the defense against GI pathogens. The mechanisms for action utilizing antimicrobial secretion, competitive exclusion from sites of adhesion and nutritional sources, improving the role of the intestinal barrier, and immunomodulation are thus used as beneficial effects on the host (167). Moreover, probiotic microbes produce microcin that works for cell entry by binding Fe-siderophore receptors or releases harmful materials after entry into the cell. These mechanisms contribute to the inhibition of intracellular enzymes like adenosine triphosphate (ATP) synthase, RNA polymerase, and deoxyribonucleic acid (DNA) gyrase, and their functions including translation into the mRNA and then induce the death of pathogenic cells (94). Several fruit juices were prepared by Wang et al. (122) such as white grape juice, pineapple juice, and orange juice, and fortified with encapsulated probiotic bacteria, *Bifidobacterium adolescentis*. The cells of *B. adolescentis* (ATCC 15703) were caught with protein-alginate capsules of faba, lentil, soy, and pea and subject to synthetic intestinal fluids (SIF, pH 6.5/37 Â°C) and gastric juice (SGJ pH 2.5/37°C). In cell counts 5.1, 5.5, 3.3, and 1.9 log cells, respectively, were *B. adolescentis* cells captured in faba, lentil, soy, and pea protein-alginate capsules after 2 h. Releases of the encapsulated cells of *B. adolescentis* in SIF over 3 h suggested that almost all cells, regardless of their wall content, were released within the first 10 min. In the pineapple and White grape juice, encapsulated *B. adolescentis* cells survived, but not in orange juice. Moreover, these fruit juices can be used as immunity boosters to avoid viral diseases such as COVID-19 (122). Recently, Mostafa et al. (192) developed the date juice containing L. sakei and L. acidophilus and examined the anti-proliferative activity against Hep-2 and Caco-2 cell lines. Total polyphenolic content and antioxidant activity were increased during storage in juice containing *L. sakie* strains. Probiotic date juice showed an anti-tumor response against the larynx Hep-2 cell linen with no response against Caco-2 cell linen (192).

Attributed to the biochemical composition, fruits and vegetables offer great advantages for improving the immune system and prevent several kinds of diseases. The encapsulation of vitamin-C and vitamin-E as well as probiotic strains not only improves the nutritional value of fruits and vegetables-based products but also boosts our immunity. Therefore, the intake of such products may protect the human body from being infected by the coronavirus. However, the blind use of traditional probiotics for COVID-19 shall not be suggested before we have a clearer idea of the pathogenesis of SARS-CoV-2 and its impact on gut microbiota.

### Dairy-Based Products

A variety of functional and nutritious products from vitamin, mineral, and bioactive fortification to promote health benefits have been supplied with dairy products as the most common deliverables (131). As milk and dairy products are part of a regular daily diet, it is expected that any new product launched will gain market share (131). There are many studies in this link on various dairy products, such as milk (136, 193, 194), yogurt (195) and cheese (196). These dairy products have been fortified with different compounds to improve versatility and provide nutrients to human health. None of them, however, concentrated on its use as an immunity booster because they had fortified dairy products with some compounds of immunological activity.

Various compounds with immunological activity, such as ω-3 fatty acids, vitamin-D, probiotics, and Fe have been encapsulated for fortifying dairy products (Table 3C). Omega (ω)-3 fatty acids in soya lecithin shrimp oil have been encapsulated for the fortification of skim milk (130). Shrimp oil nanoliposomes (SONL) fortified by 10/100 ml of skim led to a high level of bitterness. β-glucan, with 0.1 g/100 mL, was almost fully extracted from SONL by adding fortified skim milk. *In vitro* digestion studies showed that almost half of EPA and DHA were bio-accessible for body adsorption following the release of shrimp oil into the GI tract from nanoliposomes, and the microbial load of fortified skimming milk was stable over 15 days at 4°C. Such foods, high in ω-3 fatty acids, are capable of improving the human immune system discussed in the previous section (see Section Cereal-Based Products), and the formulation of these products can be used as an immunity booster for further study and recommendations for patients with COVID-19. In addition, vitamin-D is also an important precursor to a fat-soluble steroid hormone that plays a role in various body functions, including innate and adaptive immune responses. Vitamin-D also facilitates differences between macrophages and monocytes, enhancing the production of superoxide, phagocytosis, and the destruction of bacteria (24). Furthermore, vitamin-D can modulate the adaptive immune response by suppressing the cell function THP-1 and reducing the development of IL-2 and interferon-gamma proinflammatory cytokines (INF-α). Vitamin-D also activates anti-inflammatory cell cytokines by suppressing Th1 cells indirectly, diverting pro-inflammatory cells to anti-inflammatory cells (197). The frequency and severity of the COVID-19 infection have also been proposed as a deficiency in vitamin-D (25, 26, 64). Low serum vitamin-D levels were related to acute respiratory tract infections, including epidemic influenza, in clinical trials (198). Some recent findings have shown that inadequate vitamin-D will affect the role of the respiratory immune system and increase the risk of severity and mortality in COVID-19 patients (25, 199). Few observational experiments have also been performed to establish the correlation between the vitamin-D levels and the incidence and mortality of the COVID−19 levels (26, 64, 88). Vitamin-D is therefore recommended to improve immunity and reduce human mortality from COVID-19. In this regard, fortified milk and lassi (milk-based Indian beverage) developed by Golfomitsou et al. (134) and Maurya and Aggarwal (133) respectively, with soya lecithin encapsulated vitamin-D_3_ and could serve as a proper human supplement to prevent the spread of COVID-19. In both products, the vitamin-D_3_ nanoparticles demonstrated stability in the milk system and the sensory evaluation revealed their overall acceptability to customers. Additional levels of vitamin-D have been proposed to help protect the respiratory epithelium and decrease the risk of infection against pathogens (26). Vitamin-D has a beneficial impact on the immune system, which is useful for COVID-19 ARDS patients during cytokine storms. Vitamin-D supplements have also been known to help minimize viral infection incidence and severity, with a reverse association between serum levels of 25-hydroxyvitamin-D and the upper respiratory tract infection (24).

Iron-fortified yogurt, developed by Gutiérrez et al. (138) by encapsulation in sorbitan monooleate lauryl alcohol and Glycerol, may also be used against coronavirus as Fe plays an important role in our immune system (66). Fe also plays a key role in immunosurveillance through its use in the growth and differentiation of immune cells, as well as in the control of cell-mediated immune response and cytokine production. Fe deficiency results in impaired cell immunity, leading in particular to defective T-cell maturation, discontinuation of macrophage differentiation, and impaired natural killer cells (NKCs) function. The link between Fe deficiency and reduced immune function has been established as iron deficiency anemia (IDA) patients have increased morbidity due to infectious disease. It is also necessary to maintain the amount of Fe in our body (66, 68). As a result, developed Fe-fortified yogurt showed almost identical rheological and sensory properties compared to the control sample (138). This fortified yogurt will serve as an effective product to increase the Fe content of the human body and eventually enhance the immunity of our body against various viral diseases such as COVID-19. A lower concentration of serum Fe was found as an independent risk factor for death in COVID-19 patients (200). Lower body Fe and high tissue Fe levels have been associated with lower lung function and severe pulmonary inflammation, respectively (201). Further, probiotics as discussed above can be encapsulated using dairy matrices and yogurt, fermented milk and cheese have long been used as probiotic carrier dairy foods (202). During the last two decades, probiotic yogurt has been a major consumer achievement, while the solid texture, high content of fat, and pH values of cheese better preserve probiotic cells (203). Yakult—a Japan-based fermented probiotic dairy beverage is prepared from skim milk or skimmed milk powder, sugar, and glucose which is fermented by *L. casei* Shirota. It contains 8 log cfu/mL of *L casei* Shirota strains and is effective in preventing digestive disorders as well as helping building immunity and reducing the risk of infections (204, 205). Most recently, dairy waste including buttermilk whey; cheese whey; and milk or cheese whey permeate has been shown to be ideal candidates for the production of functional beverages (206).

Curcumin, another BAC, is an important antioxidant, anti-carcinogenic and anti-inflammatory agent, but sensitive to heat, light, and oxygen, and also has very low water solubility (2.99 × 10^−8^ M) (207). Its encapsulation thus makes it possible to supply the required dose of curcuminoid to the colon, where they can be easily absorbed. Curcumin has been used as a traditional medicine to treat a spectrum of diseases like rheumatism, diarrhea, intermittent fevers, hepatic disorders, inflammations, leukoderma, amenorrhea, arthritis, colitis, hepatitis, etc. (208, 209). Curcumin's immunomodulating ability is the result of its association with different immunomodulators, not just cellular elements, such as macrophages, DCs, as well as lymphocytes (B and T), but also molecular components involved in inflammatory processes, including cytokines and different transcription factors with their downstream signals (210, 211). The immunostimulation feature of DCs and interferes with the myeloid DC maturation has been found to be inhibited by curcumin. These results have been correlated with the loss of the expression of CD80 and CD86, two co-working membrane proteins that provide the motivation signal required to stimulate T cells, and impairments in the development of proinflammatory cytokine (IL-12) due to NF-κB translocation and inhibition of MAPK (Mitogen-Activated Protein Kinase) activation (212, 213). In comparison, a slightly elevated serum level of immunoglobulin M (IgM) and immunoglobulin G (IgG) curcumin in the Rabbit Diet (2, 4, and 6 g/kg), indicating a possible enhancement of the immune system in curcumin (214). Milk is an important substrate for curcumin delivery in the gut. It possesses distinct structural and physicochemical features as the binding of ions and smaller compounds, self-assembly, and surface-active traits, gelling capacity, pH-based swelling behavior, ability to regulate the matrix rehydration kinetics, the release of BACs, generation of complexes and conjugates from macromolecules' interactions, biocompatibility, and biodegradability, potential to protect encapsulated compounds, and increasing the bioaccessibility and bioavailability of functional components (215–217). In this context, the Ministry of Ayush, Government of India has advised Golden Milk as an immunity-enhancing intervention for self-care during the COVID-19 crisis (218). This milk can be prepared with a half teaspoon of turmeric powder in 150 ml of hot milk. Curcumin can alter surface protein structure in viruses and obstruct the penetration of viruses. It also affects the membrane protein by altering host lipid bilayer functionality (12, 13). The systemic inflammatory reaction of type 1 and type 2 assist cells to activate the cardiovascular symptoms in patients with COVID-19 infection (36). In the myocardial ischemia-reperfusion study, curcumin has been shown to suppress inflammation and necrotic tissues in rats by hindering early-growth response-1 and TNF-α and interleukin-6 reductions (219). Curcumin has therefore demonstrated antiviral activity against many various viruses and may be a treatment choice for COVID-19 disease control (220).

Encapsulation of the above-discussed BACs and their intake would be an ideal solution for boosting immunity to manage the COVID-19 pandemic. Omega (ω)-3 fatty acids, Fe, probiotics, curcumin, etc. can be encapsulated successfully in milk and its products and help our body to resist any kind of undesired change. However, intake of such BACs must be standardized for their getting optimal health benefits.

### Meat and Fish-Based Products

**Figure 6:**
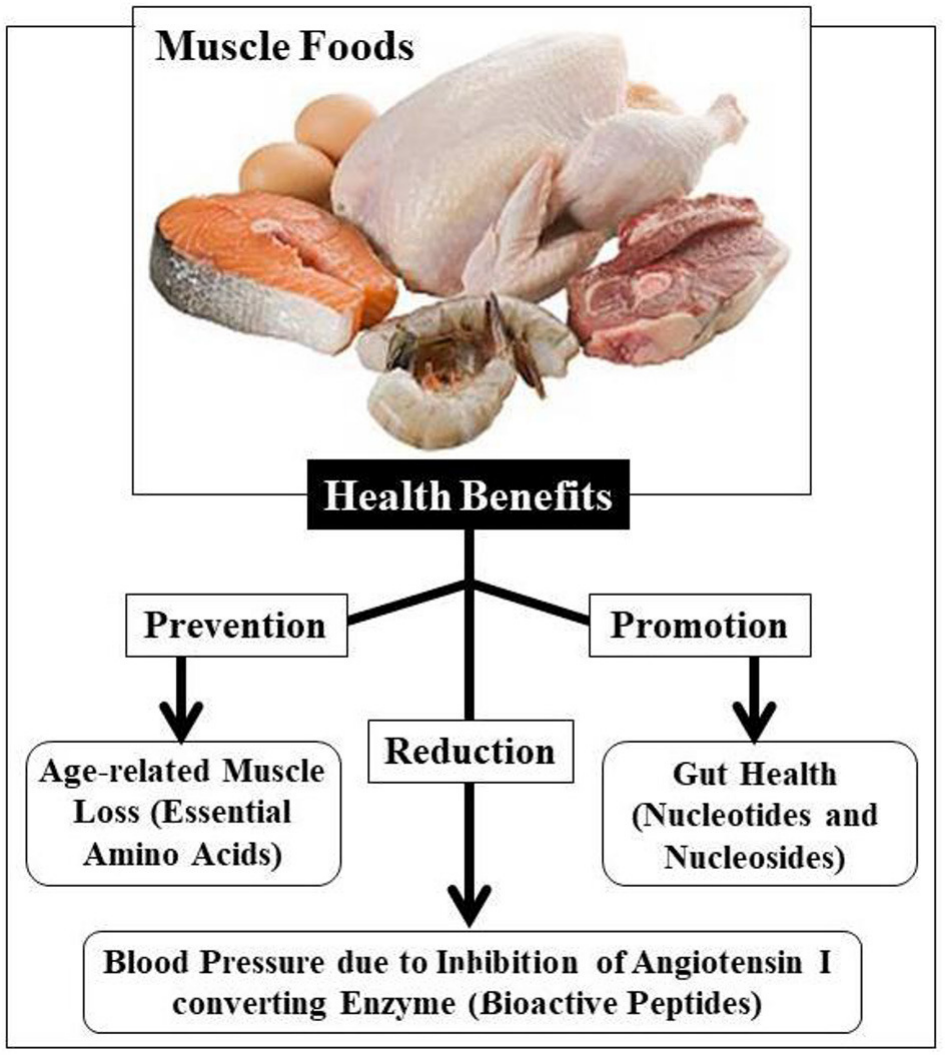
Health benefits of muscle food (221).

Muscle foods (like meat and fish) have been recognized as good sources of nutritional components, including high-quality proteins, essential amino acids (EAAs), vitamin-B complexes, minerals, and numerous other micronutrients (221–223). Numerous health advantages are linked to specific nutrients in muscle food (Figure 6). Muscle foods are known to spoil rapidly and reduce the quality and sustainability of food. Therefore, several researchers develop novel approaches to enhance muscle foods' health and shelf-life through the improvement of their nutrient profile or the addition of (natural) preservatives (221, 224, 225).

Modern nanotechnological techniques can also be applied to fortify meat and fish-based products with valuable preservatives, nutrients, or nutraceuticals (222). Generally, a wide variety of bioactive food-grade substances have been used in meat and fish-based food products for encapsulation with the application of nanotechnology-based delivery systems (6). These bioactive food-grade substances are antimicrobials, antioxidants, fat-soluble vitamins, essential oils, triglyceride oils, flavoring oils, and nutraceuticals (61, 142, 147). The integration into the meat and fish-based food products of food-grade substances by nanoemulsions affects their taste, texture, flavor, nutritional value, and shelf-life. For instance, ω-3 PUFAs have several beneficial effects on human health. The intake of fatty acids can also promote the growth and functioning of the immune system (40, 142, 222, 226). To increase ω-3 fatty acid content, Pourashouri et al. (143) introduced fish oil into fish sausage. There are some benefits to the use of fish oil-loaded nano-emulsions (as opposed to bulk oils) to enhance processed ω-3 fatty acid in meat and fish-based food products. For instance, the production of an even blend of fish oil in all meat and fish-based food products is simpler, which is clearer visually. Furthermore, due to the rapid and absolute digestion of small lipid droplets within the GI tract, the bioavailability of fish oil after ingestion may increase. The ω-3 fatty acid content of chicken meat, which contributes to improved biomarkers for human health, is enhanced by flaxseed oil-nanoemulsions, which are a rich source of plant α-linolenic acids (147). Different scientists have also confirmed that encapsulated ω-3 fatty acids have been used in nano-emulsions, for example, chicken sausage flaxseed oil (151); cod liver oil in chicken nuggets (150); Chia oil in the beef burger (149); and chia oil, linseed oil and tigernuts oil in Deer pâtés (146). The ω-3 PUFAs include docosahexaenoic and eicosapentaenoic fatty acids, considered to have beneficial effects on immunity and inflammation. Interestingly, ω-3 fatty acids prevent influenza virus replication by working against viruses. Accordingly, ω-3 fatty acids can improve the oxygenation of COVID-19 patients, similarly also said by experts of the European Society for Parenteral and Enteral Nutrition (ESPEN), although there is still no clear evidence (39, 227). However, a detailed discussion has already been made regarding the improved safety of ω-3 fatty acids (see Section Cereal-Based Products).

The use of nanoemulsions including fat-soluble vitamins (especially vitamin-E, called tocopherol), can also be integrated into the meat and fish-based food products. Feng et al. (142) encapsulated vitamin-E and incorporated it in fish-based sausages. They found that the nanoemulsions of vitamin-E retain below 500 nm in particle size when held at 4°C for 16 days, and coarse emulsions of vitamin-E increased from 4 to 6 μm by particle size. The reduced particulate size, even distribution, and tocopherol nanoemulsion stability can explain their better antioxidant activity in fish sausages. Interestingly, 250 mg/kg encapsulated nanoemulsions of vitamin-E can delay lipid oxidation and boost the consistency of fish-sausages without altering their texture characteristics during cold-stored conditions. Epidemiological studies have shown that vitamin-E deficiencies influence immune responses and infectious diseases. Vitamin-E is used to enhance T-cells, boost lymphocytes with Mitogenesis, increase IL-2 cytokine secretion, enhance NK cell function, and minimizes infection risks (35, 62, 63, 183).

Antioxidants refer to the molecules which can donate radical hydrogen (H•) radical in conjunction with other free radicals required to avoid the proliferation of oxidation reactions (228). Natural antioxidant compounds can be derived from plant matrices containing non-polar solvents, such as acetone, chloroform, ethanol, and methanol, and their combinations (229–231). The antioxidant potential of pomegranate by-products, grape seed extract, oregano, rosemary, and many different spices has been well-demonstrated in meat and poultry products (230, 232–234). The peels and seeds had more phenolic content than pulp and had the better antioxidant *in-vitro* ability. Peels and seeds are abundant in catechins, procyanidins, and hydroxycinnamic acids that encourage immunity from foreign pathogens (235, 236). Similarly, quercetin is a bioflavonoid naturally present in several vegetables and fruits quercetin supports antioxidant capacity and protects lung tissue that mainly gets damaged during infection. Laboratory and animal studies have observed that quercetin can inhibit a range of viral infections including a COVID-19-related coronavirus SARS-CoV (83). Various immune cells produce many forms of polyphenol receptors that accept and promote polyphenol cellular uptake that afterward activates signaling pathways for immune reactions (237). These compounds can (i) minimize SARS-CoV-2 viral infection by attaching to the ACE2 receptor and avoid viral infringement; and (ii) change the magnitude of COVID-19-related lung injury through ACE2 expression (238).

In the case of ginger, it has been reported that the immunity-boosting or protective activity of its components could be associated with antioxidant, antimicrobial, anti-inflammatory, and antimutagenic properties and other biological activities (239). Similarly, the major chemical constituents of tulsi are ursolic acid, oleanolic acid, eugenol, rosmarinic acid, linalool, carvacrol, and β-caryophyllene. Owing to the high concentration of eugenol, tulsi may be a COX-2 inhibitor, like many modern painkillers (240).

Thus, such encapsulated BACs will enhance our immune system. They protect us not only from influenza or bacterial infections but also from chronic or less treatable illnesses. Furthermore, eating a healthy diet cannot always be possible, therefore, integrating these bioactive encapsulated compounds into our food supply may be a potential tool for us to restore or preserve our health.

## Prospective Future and Research Opportunities

Low-immunity individuals are more vulnerable to the COVID-19 global pandemic. In this regard, the encapsulated BACs play a key function by fostering healthy bacteria in the body, which support or improve immunity (Table 1). Further, the rising desire of consumers to make healthier food choices led to a fast-growing demand for functional foods and drinks. Microencapsulation can be useful for enhancing the nutritional and health promotion of food products by adding BACs. Microencapsulation can have huge benefits to increase the supply and protection of bioprotective ingredients in food; otherwise, it cannot be made feasible. There has been a tendency for many food products to enhance immune responses, including functional foods that control the immune system by increasing or inhibiting immune response, providing host protection against infection, and reducing allergies and inflammation. Several vitamins like C, D, and E are tested for essential immune enhancement facets in addition to BACs like ω-3 fatty acids, probiotics, antioxidants, etc (Table 1). These compounds have enough potential and possibilities to be employed for the prevention of COVID-19 by boosting immunity. The effect of micronutrients on immune function has undoubtedly become an essential component of immunity. Micronutrient homeostasis preservation is an important factor in maintaining a healthy immune system and it has also shown that a variety of vitamins and trace elements play a crucial part in immune function. However, micronutrients such as vitamin-A, vitamin-B, Zn, selenium (Se), copper (Cu), magnesium (Mg) have been encapsulated but not used to develop functional foods. Potential work can be done on these compounds to develop functional foods with immunological activity. Likewise, BACs based on plants such as β-carotene, resveratrol, and other flavonoids and polyphenols have been successfully used against other viral diseases such as human immunodeficiency virus (HIV), MERS-CoV. The effect of these BACs on immunity can also be studied and used to produce functional foods using various encapsulation techniques. In this regard, the probiotics that are living microbes can provide the host with health and well-being. They play a major role in improving immunity. However, their use in the beverage sector is very limited. Even its use in other foods like meat-based foods or cereal-based food items can be explored. On the other hand, prebiotics is used to encourage wellness as a novel food product and are mostly carbohydrates and some are non-carbohydrate. Nevertheless, no studies on the prebiotic interaction mechanism for human immunity have been released. The role of prebiotics in the production of functional foods with prebiotics to enhance the immune system of the person's body is therefore important.

The production of such functional foods should be increased and commercialized to improve the immunity of the human body to various infections including COVID-19. The application and use of some compounds have been reported in different studies due to their immunological activity (Table 1). However, several other compounds have an immunological activity and have been encapsulated but surprisingly, have not been used in the formulation of any functional food. These encapsulated BACs can be recommended for use by various researchers, scientists, and industrial peoples who are concerned with food and allied subjects to develop more and more functional foods that can improve immunity in the human body, ultimately to human health. Here are many important aspects for food researchers, food scientists, and the food industry that will give them a deeper understanding of their present work and future. The majority of the encapsulated BACs observed antioxidative, antimutagenic, anti-inflammatory, properties as well as other biological activities. However, the exact component responsible for the immunity-boosting effect of such components against COVID-19 may also be studied. These important points will also raise awareness among researchers about the future of functional food and its role in the control of viral diseases such as COVID-19.

## Concluding Remarks

A healthy and strong immune system must be capable of recognizing and adapting to changes in the environment around it. Diverse micronutrients are important for immune-competence, including vitamins, trace elements, ω-3 fatty acids, probiotics, antioxidants, etc. The preservation of physical barriers of pathogens and two primary forms of immunity, both innate and adaptive immunity can contribute to varying degrees with food BACs and micronutrients. Encapsulation of these compounds with immunological activity and their use in food products can both increase the functionality and enhance human health's immunity to diseases such as COVID-19. However, far more compounds have not been investigated for their immunological effect. Therefore, researchers, scientists, and industry should concentrate on this research topic to detect and apply more immunity-enhancement compounds in the production of functional foods using encapsulation techniques to resolve the global war against COVID-19. More research with systematic approaches is needed to understand the behavior of coronaviruses and the role of food products in COVID-19 prevention.

## Author Contributions

DV has conceptualized, interpreted, corrected, and technically sound final versions of the manuscript. ST has compiled literature for manuscripts. MT, SS, AP, and MC-G have interpreted the manuscript, corrected it, and made it scientifically sound for the final version. CA and PS have provided technical suggestions, corrections, and permissions for the finalization of the manuscript. All authors contributed to the article and approved the submitted version.

## Conflict of Interest

The authors declare that the research was conducted in the absence of any commercial or financial relationships that could be construed as a potential conflict of interest.

## Abbreviations

ACE2: angiotensin-converting enzyme-2
APCs: antigen presenting cells
ARDS: acute respiratory distress syndrome
ARE: antioxidant response element
ATP: adenosine triphosphate
BACs: bioactive compounds
CCTG: caprylic/capric triglyceride
CD: β-cyclodextrin
CGC: calcium-gelatin casein
COVID-19: coronavirus disease 2019
Cu: copper
DCs: dendritic cells
DHA: docosahexaenoic acid
DNA: deoxyribonucleic acid
DPPC: di-palmitoyl phosphatidylcholine
EAAs: essential amino acids
EPA: eicosapentaenoic acid
ESPEN: European Society for Parenteral and Enteral Nutrition
Fe: iron
FSSAI: Food Safety and Standards Authority of India
GI: gastrointestinal
HACS: high amylose corn starch
HIV: human immunodeficiency virus
hs-CRP: high sensitivity C-reactive protein
IDA: iron deficiency anemia
IgA: immunoglobulin A
IgG: immunoglobulin G
IgM: immunoglobulin M
INF-α: interferon-gamma proinflammatory cytokines
LPS: lipopolysaccharide
MAPK: mitogen-activated protein kinase
MC: methyl cellulose
MD: maltodextrin
MERS-CoV: middle east respiratory syndrome–related coronavirus
Mg: magnesium
miRNA: micro RNA
mRNA: messenger RNA
NFκB: nuclear factor kappa B
NKCs: natural killer cells
Nrf2: nuclear factor erythroid 2 (nfe2)-related factor 2
PUFAs: polyunsaturated fatty acids
RNA: ribonucleic acid
ROS: reactive oxygen species
SARS-CoV-2: severe acute respiratory syndrome–related coronavirus 2
Se: selenium
SIF: synthetic intestinal fluids
SPS: Soybean protein isolates
TCR: T-cell receptor
THP: T-helper type
TLR: toll-like receptor
TNF-α: tumor necrosis factor-α
WBCs: white blood cells
WHO: World Health Organization
WPC: whey protein concentrate
WPI: whey protein isolate
Zn: zinc.
